# Dyadic effects of social support and coping on quality of life and caregiver burden among hemodialysis patient–caregiver dyads: an actor–partner interdependence mediation model

**DOI:** 10.1080/0886022X.2025.2612439

**Published:** 2026-01-12

**Authors:** LiYuan Zhang, XiaoHong Zhang, Wen Tang, Qian Wang, Li Zou, LiJuan Zhou, Yan Chen

**Affiliations:** ^a^ Department of Neurosurgery, The Affiliated Taizhou People’s Hospital of Nanjing Medical University, Taizhou, Jiangsu Province, China; ^b^ Outpatient department, The Affiliated Taizhou People’s Hospital of Nanjing Medical University, Taizhou, Jiangsu Province, China; ^c^ Nursing department, The Affiliated Taizhou People’s Hospital of Nanjing Medical University, Taizhou, Jiangsu Province, China; ^d^ Blood purification centre, The Affiliated Taizhou People’s Hospital of Nanjing Medical University, Taizhou, Jiangsu Province, China; ^e^ Endocrinology department, The Affiliated Taizhou People’s Hospital of Nanjing Medical University, Taizhou, Jiangsu Province, China; ^f^ Department of Medical Affairs, The Affiliated Taizhou People’s Hospital of Nanjing Medical University, Taizhou, Jiangsu Province, China

**Keywords:** Hemodialysis, social support, coping style, APIMeM

## Abstract

**Objective:**

This study investigates the dyadic mechanisms linking social support, coping styles, quality of life (QoL), and caregiver burden among maintenance hemodialysis (MHD) patient-caregiver dyads, with a focus on the mediating role of adaptive coping strategies.

**Method:**

In a cross-sectional design, 200 patient—caregiver dyads with MHD completed self—report measures of QoL, caregiver burden, coping style and social support. Mediation analysis was performed using actor—partner interdependence model extended to mediation (APIMeM) and the bootstrapping method. Bias-corrected 95% confidence intervals were calculated for the study of direct and indirect effects.

**Results:**

Significant actor effects were observed: patients’ social support directly associated with improved their QoL (*β* = 0.224, *p* = 0.001), while caregivers’ support associated with reduced their burden (*β* = −0.141, *p* = 0.001). Adaptive coping mediated 46.2% of patient QoL variance *via* actor effects (total indirect *β* = 0.288) and 25.0% of caregiver burden reduction *via* actor effects (total indirect *β* = −0.047). Partner effects revealed cross-dyad influences: caregivers’ support associated with enhanced patient QoL (*β* = 0.239, *p* = 0.002), and patients’ support associated with reduced caregiver burden (*β* = −0.475, *p* = 0.001). Key mediation pathways included caregivers’ support affecting patient QoL *via* caregivers’ coping (*β* = 0.050, 95% CI [0.005, 0.103]) and patients’ support affecting caregiver burden *via* caregivers’ coping (*β* = −0.164, 95% CI [−0.236, −0.103]).

**Conclusions:**

Enhancing social support networks and fostering adaptive coping strategies through dyad-centered programs may synergistically alleviate caregiver burden and improve patient QoL.

## 1. Background

Chronic kidney disease (CKD) represents a critical global health challenge [1]. To quantify its impact, the 2021 Global Burden of Disease report indicates19.94 million new cases of CKD worldwide, 673.72 million patients with CKD, 44.45 million disability-adjusted life years (DALYs) caused by CKD, and 1.53 million deaths [2]. End-stage renal disease (ESRD) is the end-stage of CKD and requires lifelong renal repla [1,3] cement therapy to maintain vital organ function [4,5]. While maintenance hemodialysis (MHD) sustains life for over 80% of ESRD patients [4], this treatment paradigm introduces new challenges. The rigorous regimen of the frequent weekly dialysis to the purification center, combined with severe dietary and fluid restrictions, physical activity, and medications [5,6], MHD patients and their caregivers experience considerable physical and psychological burdens [7,8]. MHD patients frequently experience fatigue, depression, and diminished quality of life (QoL) [9,10], while caregivers endure financial strain, role conflicts, and emotional exhaustion [11]. Building on this evidence, studies of chronic disease dyads consistently demonstrate bidirectional burden transmission [12,13]. This interaction highlights the need to explore the mechanisms of social support and coping styles from a dyadic perspective.

Recent studies suggest that social support exerts a protective effect on QoL for both patients and caregivers, yet its pathways differ [14–16]. For patients, social support alleviates anxiety and directly enhances QoL [17]. For caregivers, social support indirectly reduces burden by strengthening caregiving experience and positive emotions [18]. However, some studies reveal a “dose effect” of social support: excessive support may foster patient dependency, undermining self-management abilities [19]. These contradictory findings underscore the necessity of introducing mediating variables (e.g. coping styles) to explain complex mechanisms. Related research shows that positive coping styles make emotional responses more balanced, reduce the negative effects of the disease, and help improve QoL and adaptation to the disease [20,21]. Those who choose to deny, disengage, or avoid stressful situations (i.e. using negative coping strategies) tend to have lower perceived self-efficacy and greater distress [22]. However, no unified conclusion has been reached on the mediating effect of coping style in MHD patients and their family caregivers. Most studies used a single-level analysis (only patient or caregiver) and ignored the interdependence of coping styles in the patient-caregiver dyads. Therefore, this study clarified the relationship between social support, coping style and QoL of patients and caregiver burden in hemodialysis patient and caregiver dyads and provided a basis for the design and implementation of evidence-based mental health promotion measures in the future.

## 2. Methods

### 2.1. Design and participants

This study used a cross-sectional design and followed the STROBE guidelines [23]. The study was conducted at the Blood Purification Center of a Grade III Class A hospital in Jiangsu Province, China. Between October 2023 and November 2023, participants were selected by convenience sampling among patients undergoing MHD and their primary family caregivers. The inclusion criteria for patients were as follows: (i) meet the diagnostic criteria for CKD; (ii) MHD for more than 3 months; (iii) being aged ≥ 18 years; (iv) having a family caregiver as their partner in a dyad in this study. Family caregivers were included if they were: (i) being aged ≥ 18 years; (ii) responsible for the main care tasks of MHD patients. MHD dyads were excluded if: (i) either dyad member had a serious mental, visual, or hearing impairment that prevents normal communication; (ii) accompanied by serious diseases of vital organs. Based on the sample size principle of structural equation model [24], the sample size of this study was set to 200 dyads (200 patients and 200 caregivers) without data loss.

### 2.2. Study hypotheses

**Figure 1.**
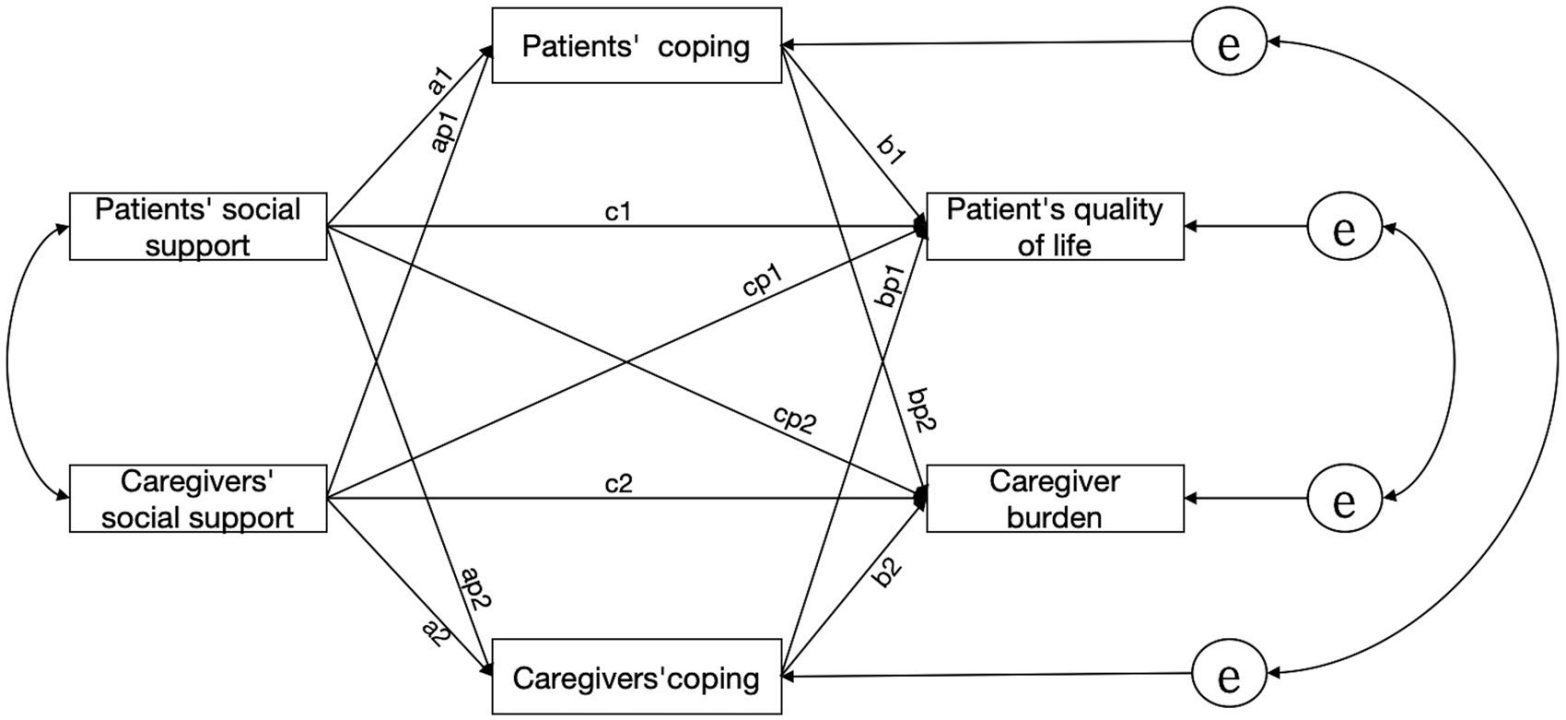
APIMeM actor—partner interdependent mediation hypothesis model. Note: Path labels (e.g., a1, ap2, b1, bp2, c1, cp2) denote statistical coefficients in the APIMeM framework without inherent conceptual meaning.

Considering the points, according to the actor—partner interdependence model extended to mediation (APIMeM) [25], MHD patients and caregivers can be regarded as distinguishable pair-wise relationships according to their roles. In this study, the social support of MHD patients and their caregivers was set as the independent variable, the coping style (adaptive coping/maladaptive coping) of them was set as the mediating variable, and the QoL of patients and caregiver burden were set as the dependent variable. An APIMeM model that could distinguish paired data was constructed and all possible relationships between variables were explored. Based on this, we propose 3 hypotheses, and the figure of the hypothetical model is shown in Figure 1.

Hypothesis 1: Social support will be positively associated with patients’ QoL, and caregiver burden, respectively.

Hypothesis 2: Coping style mediates the actor effects in the relationship between social support and QoL in patients, and between social support and caregiver burden in caregivers.

Hypothesis 3: Coping style mediates the partner effects in the relationship between caregivers’ social support and patients’ QoL, and between patients’ social support and caregiver burden.

### 2.3. Procedure

Participants were recruited from two blood purification centers of a general hospital in Taizhou, Jiangsu Province of China from June to September 2023. The principal investigator (LYZ) communicated with potential patients who meet the inclusion and exclusion criteria and recruited them after they signed the written informed consent. The questionnaires were distributed among the volunteers. The researchers were available to resolve any questions from the respondents. The completed questionnaires were returned to the researchers.

### 2.4. Measures

The Chinese version of KDQOL-36^™^ was downloaded from RAND Corporation website (https://www.rand.org/health-care/surveys_tools/kdqol.html), imported by Amgen and translated by the MAPI Institute. It can be subdivided into 5 subscales, including the SF-12 Physical Health Composite (SF-12 PC), the SF-12 Mental Health Composite (SF-12 MC), the Kidney Disease Symptoms/Problems (SP), the Burden of Kidney Disease (BKD) and the Effects of Kidney Disease (EKD). Each subscale focuses on a different aspect of the patient’s life. In the KDQOL-36TM scale, the first 2 subscales are common to the SF-12 scale and cover a wide range of health-related QoL areas. The last 3 subscales are specific to kidney disease. Each item is set to 5–6 different response levels, and patients make choices according to their actual situation. Each dimension is scored on a scale of 0–100, with higher scores indicating better QoL. The Chinese version of KDQOL-36^™^ has good reliability and validity, with Cronbach’s alpha coefficient of each dimension ranging from 0.69–0.78 [26].

The ZBI was originally developed by Zarit’s team in 1980 [27]. In 2005, it was sinicized by Wang Lie team of China Medical University [28]. It is the most used tool to assess caregiver burdecn and has been revised and improved to form a 22-item ZBI scale, which is divided into two dimensions: personal burden and responsibility burden. Each entry is rated on a scale of 0–4 points (i.e. total score 0–88). According to the total score, the burden is divided into four levels: 0–20 is rated as no or slight burden, 21–40 is rated as light to moderate burden, 41–60 is rated as moderate to heavy burden, and 61–88 is rated as heavy burden. The Chinese version of ZBI has good reliability and validity, with Cronbach’s alpha coefficient of 0.87 [29].

The Brief COPE Scale (BCS) was first developed by Carver [30] and later translated into Chinese by Kristen and Christian from the Chinese University of Hong Kong [31]. It consists of two factors: adaptive coping and maladaptive coping [32]. There are 28 items in total, each of which uses a Likert − 4 scale scoring method. “1” represents “never” to “4” representing “often”, and the higher the average score of the items, the more likely the respondent is to use this coping strategy. Cronbach’s alpha coefficient is 0.83.

The Medical Outcomes Study Social Support Survey (MOS-SSS) was developed by Sherboume [33] in the United States and translated into Chinese by Doris [34]. The scale consists of 20 items and is divided into four dimensions. They are Tangible Support, Informational and Emotional, Positive Social Interaction, and Affectionate Support. Among the 20 items, item 1 is a subjective question designed to measure the size of the patient’s support network. The remaining items are objective questions, which are scored differently based on the frequency or intensity of occurrence. Each item uses a Likert-5 scale scoring method, with “1” representing “never” to “5” representing “always”. The higher the score, the better the medical social support. The Cronbach’s alpha coefficient of the scale is 0.79.

### 2.5. Data analysis

All data were statistically analyzed using IBM SPSS software (version 29.0, International Business Machines Corp, NY, USA) and Amos software (version 23.0, International Business Machines, New York, NY, USA) with a significance level set at 5% (two-tailed). The Q-Q plot was used to test the normality of continuous data, and the mean (standard deviation) was used to describe normally distributed quantitative data. Non-normal distributed data was described using the median (interquartile range). Count data was described using frequency (percentage). Hypotheses were tested using an Actor-Partner Interdependence Model (APIM). This model takes into account the interdependence between dyad members. Specifically, it allows testing the effect of each partner on their own (an actor effect) and their partner’s outcome (a partner effect) [35]. To test whether coping style mediates social support, patients’ QoL and caregiver burden we utilized the Actor-Partner Interdependence Model extended to Mediation (APIMeM) [25] by Amos 23.0. The macro estimates dyadic mediation models with one or several mediators. The biased-corrected bootstrap method was used to resample 2,000 times for 95% confidence interval to estimate the mediation effects. If the confidence interval did not include zero, the mediation effect was considered to hold.

### 2.6. Ethical considerations

The study design and procedures followed the Declaration of Helsinki and were approved by the Ethics Committee of Taizhou People’s Hospital (KY2023-102-01). Written informed consent was obtained from each participant who agreed to participate in the study. Participants have the right to withdraw from the study at any time without any negative impact on their usual treatment, care, and other services.

## 3. Results

### 3.1. Sample characteristics

The cohort of MHD patients had a mean age of 52.79 years (SD = 12.08), with males predominating (63.0%, *n* = 126). Educational attainment analysis revealed that junior high school education ­represented the largest proportion (42.0%, *n* = 84). Most participants were married and cohabiting (87.5%, *n* = 175), while only 13.0% (*n* = 26) maintained full-time employment. Economically, 43.5% (*n* = 87) reported a monthly income below 1,000 RMB, with primary financial support derived from pensions/salaries (46.5%) or family members (29.0%). Family caregivers exhibited comparable demographic profiles, with a mean age of 52.67 years (SD = 12.87). Notably, 64.5% (*n* = 129) were aged ≥50 years, and females constituted the majority (64.5%, *n* = 129). Marital stability was observed in 91.5% (*n* = 183), and 70.0% (*n* = 140) reported stable income sources. Spousal relationships accounted for 66.0% (*n* = 132) of caregiving dyads. Comprehensive demographic data are presented in Table 1.

**Table 1.** Socio-demographic and clinical characteristics.

Characteristics	(*n* = 200)	Characteristics	(*n* = 200)
MHD patients		Caregivers	
Age	52.79 ± 12.08	Age	52.67 ± 12.87
Sex		Sex	
Men	126 (63.0%)	Men	71 (35.5%)
Women	74 (37.0%)	Women	129 (64.5%)
Education level		Education level	
Primary school or less	31 (15.5%)	Primary school or less	43 (21.5%)
Junior high school	84 (42.0%)	Junior High School	71 (35.5%)
High school	51 (25.5%)	High school	47 (23.5%)
College degree or above	34 (17.0%)	College degree or above	39 (19.5%)
Marital status		Marital status	
Unmarried	12 (6.0%)	Unmarried	11 (5.5%)
Married	175 (87.5%)	Married	183 (91.5%)
Divorced	12 (6.0%)	Divorced	4 (2.0%)
Widowhood	1 (0.5%)	Widowhood	2 (1.0%)
Employment		Employment	
Employed	26 (13.0%)	Employed	89 (44.5%)
Unemployed	28 (14.0%)	Unemployed	5 (2.5%)
Retirement	68 (34.0%)	Retirement	51 (25.5%)
Freelancer	31 (15.5%)	Freelancer	26 (13.0%)
Farming	47 (23.5%)	Farming	29 (14.5%)
Monthly income		Monthly income	
Less than 1,000 RMB	87 (43.5%)	Less than 1,000 RMB	42 (21.0%)
1,000–3,000 RMB	54 (7.0%)	1,000-3,000 RMB	60 (30.0%)
3,000–5,000 RMB	32 (16.0%)	3,000-5,000 RMB	57 (28.5%)
More than 5,000 RMB	27(13.5%)	More than 5,000 RMB	41 (20.5%)
Financial source		Financial source	
Salary/pension	93 (46.5%)	Salary/pension	144 (72.0%)
Saving	8 (4.0%)	Saving	8 (4.0%)
Supply of family members	58 (29.0%)	Supply of family members	26 (13.0%)
Relatives/friends supply	5(2.5%)	Relatives/friends supply	4 (2.0%)
Government assistance	12 (6.0%)	Government assistance	4 (2.0%)
Others	24 (12.0%)	Others	14 (7.0%)
Medical payment method		Relationship with patients	
Rural medical insurance	27 (13.5%)	Children	39 (19.5%)
Social medical insurance	171 (85.5%)	Spouse	132 (66.0%)
Self-financing	2 (2.0%)	Parent	5 (12.5%)
Dialysis time		Others	24 (12.0%)
Less than 2 years	59 (29.5%)		
2–4 years	49 (24.5%)		
4–10 years	60 (30.0%)		
More than 10 years	32 (16.0%)		

### 3.2. Descriptive statistics

Descriptive statistics (i.e. means and standard deviations) and correlations of the study variables are presented in Table 2. For patients, QOL was significantly positively correlated with social support and adaptive coping, and conversely, was significantly negatively correlated with maladaptive coping and caregiver burden. For caregivers, caregiver burden had a significant negative correlation with social support and adaptive coping, and a significant positive correlation with maladaptive coping.

**Table 2.** Means, standard deviations, and correlations of the study variables.

Variables	Patients’ QoL	Patients’ social support	Patients’ adaptive coping	Patients’ maladaptive coping	Caregiver burden	Caregivers’ social support	Caregivers’ adaptive coping	Caregivers’ maladaptive coping
Patients’ QoL	–							
Patients’ social support	0.640**	–						
Patients’ adaptive coping	0.685**	0.666**	–					
Patients’ maladaptive coping	−0.381**	−0.509**	−0.253**	–				
Caregiver burden	−0.638**	−0.714**	−0.497**	0.502**	–			
Caregivers’ social support	0.458**	0.306**	0.310**	−0.303**	−0.380**	–		
Caregivers’ adaptive coping	0.457**	0.461**	0.390**	−0.285**	−0.669**	0.251**	–	
Caregivers’ maladaptive coping	−0.360**	−0.416**	−0.266**	0.451**	0.565**	−0.272**	−0.514**	–
M	66.54	65.36	2.38	2.04	41.21	62.10	2.46	2.01
SD	14.55	15.67	0.53	0.56	14.51	13.76	0.66	0.63

* *p* < 0.05. **p < 0.01.

### 3.3. Mediation analysis

**Figure 2.**
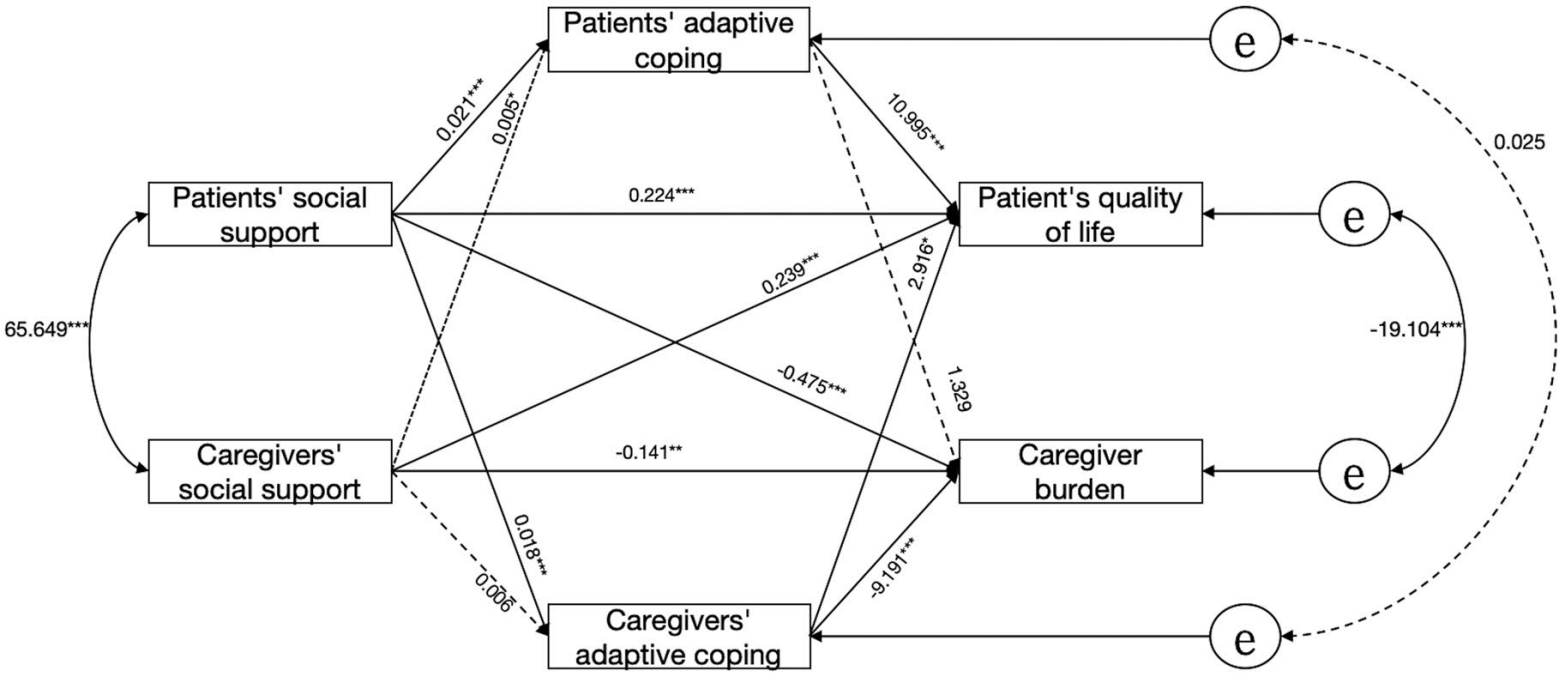
APIMeM mediation analysis of adaptive coping. Note: *p < 0.05. **p < 0.01

We explored the relationship of all variables using adaptive coping and maladaptive coping as a mediating variable separately. Parameter estimation employed maximum likelihood methodology, with equality constraints imposed on actor/partner effects to determine whether actor and partner effects were equal (a1 = a2; ap1 = ap2; b1 = b2; bp1 = bp2; c1 = c2; cp1 = cp2). Model comparison revealed significant divergence between the restricted and saturated models (*χ*^2^ = 295.652, *p* < 0.001), rejecting the hypothesis of equivalent actor-partner effects. Subsequent analysis using the Actor-Partner Interdependence Mediation Model (APIMeM) quantified pathway coefficients, with final actor-partner interdependence mediation diagram is shown in Figure 2, and the direct and indirect effects of social support on QoL and caregiver burden are shown in Table 3.

**Table 3.** Mediation path coefficients for APIMeM of adaptive coping (*N* = 200).

Effect	Path	Effect size	95%CI		*P*
			upper	lower	
Actor effect					
Patient					
Total effect		0.512	0.419	0.611	0.001
Total indirect effect		0.288	0.206	0.390	0.001
Actor-actor single indirect effect	Patients’ social support—patients’ adaptive coping—patients’ QoL	0.236	0.154	0.333	0.001
Partner-partner single indirect effect	Patients’ social support—caregivers’ adaptive coping—patients’ QoL	0.052	0.017	0.103	0.004
Direct effect	Patients’ social support—patients’ QoL	0.224	0.109	0.346	0.001
Caregiver					
Total effect		−0.188	−0.284	−0.09	0.001
Total indirect effect		−0.047	−0.110	0.003	0.064
Actor-actor single indirect effect	Caregivers’ social support—caregivers’ adaptive coping—caregiver burden	0.006	−0.008	0.031	0.346
Partner-partner single indirect effect	Caregivers’ social support—patients’ adaptive coping—caregiver burden	−0.053	−0.116	−0.001	0.038
Direct effect	Caregivers’ social support—caregiver burden	−0.141	−0.224	−0.06	0.001
Partner effect					
Patient					
Total effect		0.306	0.179	0.437	0.001
Total indirect effect		0.067	0.016	0.123	0.016
Actor-partner single indirect effect	Caregivers’ social support—caregivers’ adaptive coping -patients’ QoL	0.05	0.005	0.103	0.036
Partner-actor single indirect effect	Caregivers’ social support—patients’ adaptive coping—patients’ QoL	0.017	0.002	0.048	0.021
Direct effect	Caregivers’ social support—patients’ QoL	0.239	0.112	0.366	0.002
Caregiver					
Total effect		−0.611	−0.705	−0.514	0.001
Total indirect effect		−0.136	−0.224	−0.040	0.005
Actor-partner single indirect effect	Patients’ social support—patients’ adaptive coping—caregiver burden	0.028	−0.048	0.103	0.482
Partner-actor single indirect effect	Patients’ social support—caregivers’ adaptive coping—caregiver burden	−0.164	−0.236	−0.103	0.001
Direct effect	Patients’ social support—caregiver burden	−0.475	−0.599	−0.358	0.001

In terms of the actor effect, the direct effect was significant. Patients’ social support was significantly associated with better their own QoL (*β* = 0.224, *p* = 0.001), and caregivers’ social support showed a significant negative association with caregiver burden (*β* = −0.141, *p* = 0.001). The indirect effect was significant, the social support of patients could indirectly and positively predict the QoL of patients through the adaptive coping of patients or caregivers (*β*_patient_ = 0.236, *p* = 0.001; *β*_caregiver_ = 0.052, *p* = 0.004). Caregivers’ social support could negatively predict caregivers’ burden through patients’ adaptive coping (*β* = −0.053, *p* = 0.038). However, the indirect effect of the actor-actor of caregivers was not significant (*β* = 0.006, *p* = 0.346). In terms of the partner effect, caregiver social support could directly positively predict the QoL of patients (*β* = 0.239, *p* = 0.002), and patient social support could directly negatively predict caregiver burden (*β* = −0.475, *p* = 0.001). Caregivers’ social support could affect patients’ QoL through adaptive coping (*β*_caregivers_ = 0.050, *p* = 0.036; *β*_patient_ = 0.017, *p* = 0.021), and the indirect effect of patient was significant. Among the indirect effects of caregivers, patients’ social support could negatively predict caregivers’ burden through caregivers’ adaptive coping (*β* = −0.164, *p* = 0.001).

The biased-corrected bootstrap method was used to resample 2,000 times for 95% confidence interval estimation to test the mediating effect of adaptive coping between social support of both patients and caregivers and their QoL and caregiver burden. If the confidence interval did not include zero, the mediation effect was considered to hold. The results showed that social support affected the QoL of MHD patients through adaptive coping of both sides (*β* = 0.236, 95%CI [0.154, 0.333]; *β* = 0.052, 95%CI [0.017, 0.103]), the mediating effect was significant. Caregivers’ social support affected caregivers’ burden through patients’ adaptive coping (*β* = −0.053, 95%CI [−0.116, 0.001]), and the mediating effect was significant. Caregivers’ social support affected patients’ QoL through adaptive coping of both sides (*β* = 0.050, 95%CI [0.005, 0.103]; *β* = 0.017, 95%CI [0.002, 0.048]), and the mediating effect of the partner effect was significant. Social support of patients affected caregiver burden through caregiver adaptive coping (*β* = −0.164, 95%CI [−0.236, 0.103]), the mediating effect of the partner effect was significant. However, caregivers’ social support could not affect caregivers’ burden through their own adaptive coping (*β* = 0.006,95%CI [−0.008, 0.031]). Patients’ social support could not affect caregivers’ burden through patients’ adaptive coping (*β* = 0.028,95%CI [−0.048, 0.103]).

**Figure 3.**
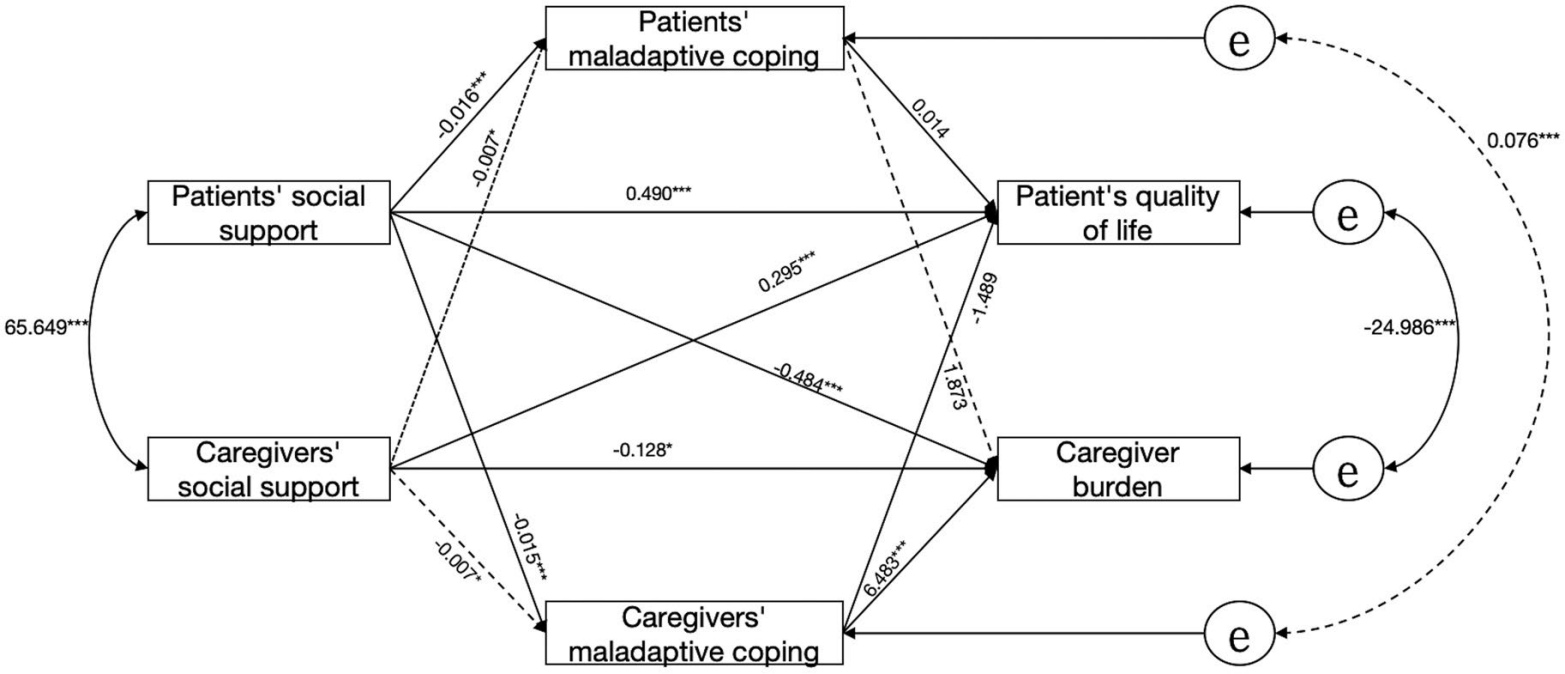
APIMeM mediation analysis of maladaptive coping. Note: *p < 0.05. **p < 0.01

The results of the analysis of maladaptive coping as a mediating variable were slightly different from those of adaptive coping, in that neither the social support of the patient nor the caregiver could improve the quality of life of the patient through maladaptive coping on both sides (*β* = 0.000, 95%CI [−0.061, 0.065]; *β* = 0.022, 95%CI [−0,021, 0.067]; *β* = 0.011, 95%CI [−0.014, 0.043]; *β* = 0.000, 95%CI [−0.028, 0.003]). The actor and partner total indirect effects of patients were not significant, and other pathways and adaptive coping were similar. Detailed details are provided in Figure 3 and Table 4.

**Table 4.** Mediation path coefficients for APIMeM of maladaptive coping (*N* = 200).

Effect	Path	Effect size	95%CI		*P*
			upper	lower	
Actor effect					
Patient					
Total effect		0.512	0.419	0.611	0.001
Total indirect effect		0.022	−0.033	0.083	0.394
Actor-actor single indirect effect	Patients’ social support—patients’ maladaptive coping—patients’ QoL	0.000	−0.061	0.065	0.977
Partner-partner single indirect effect	Patients’ social support—caregivers’ maladaptive coping—patients’ QoL	0.022	−0.021	0.067	0.271
Direct effect	Patients’ social support—patients’ QoL	0.49	0.387	0.597	0.001
Caregiver					
Total effect		−0.188	−0.284	−0.09	0.001
Total indirect effect		−0.06	−0.126	−0.014	0.009
Actor-actor single indirect effect	Caregivers’ social support—caregivers’ maladaptive coping—caregiver burden	−0.012	−0.039	0.005	0.166
Partner-partner single indirect effect	Caregivers’ social support—patients’ maladaptive coping—caregiver burden	−0.047	−0.105	−0.008	0.011
Direct effect	Caregivers’ social support—caregiver burden	−0.128	−0.228	−0.034	0.009
Partner effect					
Patient					
Total effect		0.306	0.179	0.437	0.001
Total indirect effect		0.011	−0.014	0.043	0.343
Actor-partner single indirect effect	Caregivers’ social support—caregivers’ maladaptive coping -patients’ QoL	0.011	−0.006	0.047	0.182
Partner-actor single indirect effect	Caregivers’ social support—patients’ maladaptive coping—patients’ QoL	0.000	−0.028	0.03	0.983
Direct effect	Caregivers’ social support—patients’ QoL	0.295	0.163	0.428	0.001
Caregiver					
Total effect		−0.611	−0.705	−0.514	0.001
Total indirect effect		−0.127	−0.212	−0.059	0.001
Actor-partner single indirect effect	Patients’ social support—patients’ maladaptive coping—caregiver burden	−0.031	−0.088	0.014	0.175
Partner-actor single indirect effect	Patients’ social support—caregivers’ maladaptive coping—caregiver burden	−0.096	−0.162	−0.05	0.001
Direct effect	Patients’ social support—caregiver burden	−0.484	−0.602	−0.368	0.001

## 4. Discussion

This study extends prior evidence [1] by delineating the distinct mechanisms through which social support influences QoL and caregiver burden among MHD patient-caregiver dyads, with adaptive coping serving as a critical mediator. Our findings corroborate the actor-partner interdependence framework [25] while revealing novel cross-dyad pathways that challenge conventional unidirectional models of chronic disease management.

The significant actor effects align with stress-buffering theories [36], where patients’ perceived social support was directly associated with better QoL (*β* = 0.224, *p* = 0.001)—a finding consistent with CKD populations in Western settings [17,37,38]. Notably, caregivers’ social support exhibited dyadic functionality: being directly associated with reduced burden among themselves (*β* = −0.141, *p* = 0.001) while showing indirect associations with better patient QoL through two cross-dyad coping pathways:(a) *via* caregivers’ own adaptive coping (*β* = 0.050, *p* = 0.036) and (b) *via* patients’ adaptive coping (*β* = 0.017, *p* = 0.021). This bidirectional resource transfer mirrors recent findings in hemodialysis [39], disabilities [40], and couples [40,41] dyads. Specifically, supportive coping interventions for caregivers, including multi-modal strategies such as health literacy modules, symptom management toolkits, and peer support networks, may improve caregiving competence and perceived support levels, thereby improving both caregiver burden and patient QoL.

The partner effects revealed unexpected potency: patients’ social support demonstrated stronger burden reduction in caregivers (*β* = −0.475) than caregivers’ own support systems. This contradicts the “caregiver-centric” intervention paradigm and implies that empowering *patients’* social networks may yield disproportionate benefits for caregiver well-being—a phenomenon potentially amplified in collectivist cultures prioritizing familial reciprocity [42]. As the illness duration of MHD patients prolongs, their self-care capacity typically diminishes, leading to increasingly complex and multifaceted caregiving demands that exacerbate caregiver burden. Compared with those targeting only the patient or caregiver, interventions that approach the family dyadic perspective while considering the mutually supportive relationship and interaction between the caregiver and patient are expected to benefit both parties [43,44]. The observed strong partner effects likely reflect Confucian values of familial reciprocity that are central to Chinese caregiving contexts. Caution should be exercised when generalizing these findings to individualistic cultural settings without empirical cross-validation.

The analysis of the actor-partner interdependence mediation found that, adaptive coping mediated 46.2% of patient QoL variance (total indirect *β* = 0.288) but only 25.0% of caregiver burden reduction (total indirect *β* = −0.047), indicating: patients’ coping strategies served as primary mediators (*β* = 0.236 vs. caregiver *β* = 0.052), supporting the patient as active agent in MHD adaptation. Despite higher maladaptive coping scores (*M* = 2.01 vs. patient *M* = 2.04, Table 2), caregivers’ self-mediation was non-significant (*β* = 0.006, 95% CI [−0.008, 0.031]). Our correlational analyses revealed significant dyadic interdependence between patients’ and caregivers’ coping strategies (*r* = 0.390, *p* < 0.01; Table 2), previous research corroborates this notion [45]. Building on longitudinal evidence from prior research [44], which demonstrated that patients’ adaptive coping improvements temporally preceded caregivers’ corresponding changes. The findings suggest a potential cascading effect wherein caregivers’ coping strategies may be positively influenced by patients’ prior psychological adaptation, which is similar to the findings of current study [46]. This sequential pattern implies that early interventions targeting patients’ coping skills could create secondary benefits for caregiver adjustment through dyadic interaction processes. This is consistent with the traditional perception of Chinese families that caregivers prioritize patient needs over personal coping.

### 4.1. Limitations and future directions

Three critical limitations warrant cautious interpretation of our findings. First, the data collected in this study relied solely on self-report measures, which may introduce temporal ambiguity and the accuracy of the reported information. In addition, our reliance on cross-sectional data prevents us from making causal claims about the observed associations and limits our ability to examine changes over time. Third, we used a single-center convenience sampling method, which may limit the representativeness of the sample. Future studies should adopt an experimental design, add objective observational measures, and expand the range of samples in multiple centers. Longitudinal studies with long-term follow-up of of participants and longitudinal modeling analyses would provide more robust insights into the temporal associations and causal relationships among social support, coping, QoL, and caregiver burden.

## 5. Conclusions

This study pioneers the application of APIMeM in MHD dyads, revealing that social support operates not merely as a personal resource but as a relational currency transmitted through adaptive coping pathways. By quantifying both actor and partner effects, we provide an empirical foundation for dyad-centered interventions that synergistically enhance patient QoL and mitigate caregiver burden. Future research should explore temporal dynamics of these pathways across different cultural and socioeconomic contexts.

## Data Availability

The authors are willing to permanently share data supporting the results and analysis presented in the paper after publication. The data can be used for scientific research beneficial to human health. The datasets generated during and/or analyzed during the current study are available from the corresponding author on reasonable request.
